# Temporo-spatial dynamics and behavioural patterns of 2012 cholera epidemic in the African mega-city of Conakry, Guinea

**DOI:** 10.1186/s40249-018-0393-8

**Published:** 2018-02-15

**Authors:** Alexandre Blake, Veronique Sarr Keita, Delphine Sauvageot, Mamadou Saliou, Berthe Marie Njanpop, Fode Sory, Bertrand Sudre, Koivogui Lamine, Martin Mengel, Bradford D. Gessner, Keita Sakoba

**Affiliations:** 1 Agence de Médecine Préventive, Paris, France; 2 Division Prévention et Lutte contre la Maladie (DPLM), Ministère de la santé Publique et de l’Hygiène Publique Conakry, Conakry, Guinea; 3 Institut National de Sante Publique (INSP), Conakry, Guinea; 4 Franche-Comté university (UMR 6249 chrono-environnement), Besançon, France

**Keywords:** Cholera, Space-time clustering, Guinea

## Abstract

**Background:**

Cholera is endemic in Guinea, having suffered consecutive outbreaks from 2004 to 2008 followed by a lull until the 2012 epidemic. Here we describe the temporal-spatial and behavioural characteristics of cholera cases in Conakry during a three-year period, including the large-scale 2012 epidemic.

**Methods:**

We used the national and African Cholera Surveillance Network (Africhol) surveillance data collected from every cholera treatment centre in Conakry city from August 2011 to December 2013. The prevalence of suspect and confirmed cholera cases, the case fatality ratio (CFR), and the factors associated with suspected cholera were described according to three periods: pre-epidemic (A), epidemic 2012 (B) and post epidemic (C). Weekly attack rates and temporal-spatial clustering were calculated at municipality level for period B. Cholera was confirmed by culture at the cholera national reference laboratory.

**Results:**

A total of 4559 suspect cases were reported: 66, 4437, and 66 suspect cases in periods A, B and C, respectively. Among the 204 suspect cases with culture results available, 6%, 60%, and 70% were confirmed in periods A, B, and C, respectively. With 0.3%, the CFR was significantly lower in period B than in periods A (7.6%) and C (7.1%). The overall attack rate was 0.28% in period B, ranging from 0.17% to 0.31% across municipalities. Concomitantly, a cluster of cases was identified in two districts in the northern part of Conakry. At 14%, rice water stools were less frequent in period A than in period B and C (78% and 84%). Dehydration (31% vs 94% and 89%) and coma (0.4% vs 3.1% and 2.9%) were lower during period B than in periods A and C. The treatment of drinking water was less frequent in period A, while there were more reports of recent travel in period C.

**Conclusions:**

The epidemic dynamic and the sociological description of suspect cases before, during, and after the large-scale epidemic revealed that the *Vibrio cholerae* was already present before the epidemic. However, it appeared that infected individuals reacted differently in terms of disease severity as well as their access to treated water and travel habits. Such an in-depth description of cholera epidemics should be systematically carried out in cholera endemic settings in order to prioritize higher risk areas, identify transmission factors, and optimize preventive interventions.

**Electronic supplementary material:**

The online version of this article (10.1186/s40249-018-0393-8) contains supplementary material, which is available to authorized users.

## Multilingual abstracts

Please see Additional file 1 for translations of the abstract into the five official working languages of the United Nations.

## Article summary line

During the 2012 cholera outbreak in Conakry, the Space-Time clustering context — the combination of the geographical impact of the disease, predominantly in the northern part of the city, and the variation of behavioural patterns over a broad timeframe — highlighted this zone as a potential hotspot; its mobile population, limited access to clean water, and inadequate sanitation all suggested that an intervention in this area would have the biggest public health impact.

## Background

During 2003–2013, Guinea experienced 25 358 cholera cases [1–13], mainly due to annual outbreaks from 2004 to 2008, followed by a lull until a major outbreak during 2012. The country’s capital Conakry accounted for 45% of notified cholera cases during this period [14]. The 2012 outbreak accounted for 7350 clinical cases, of which 4437 (60%) were in Conakry [11]. The number of cholera cases in Conakry before and during 2012 spiked after an earlier rise in cases in coastal areas, raising the possibility that local or transient fishermen were triggering outbreaks. This hypothesis has not, however, been evaluated [14, 15]. Similarly, despite some reports from the 2012 outbreak, formal temporal, spatial, and risk factor analyses have not been published [16].

In severely resource-limited areas such as Guinea, identifying transmission patterns and geographic foci of cholera will help lead to more efficient interventions, including water sanitation, and vaccine campaigns. However, surveillance capacity is also limited, which in turn leads to a lack of data or inaccurate data. For example, cholera may present with similar clinical and epidemiological features as Ebola [17–19], which emphasizes the need for quality data in a country such as Guinea where both diseases might have co-existed. In this context, the African Cholera Surveillance Network (Africhol), associated to the Guinean Ministry of Health (MoH), established enhanced prospective cholera surveillance in Conakry in August 2011. Based on data collected at this site, we describe here the evolution of the 2012 cholera outbreak in Conakry, including clinical, behavioural, and microbiological information.

This limitation in resources and the social, societal, and environmental challenges are common to many African cities. A large part of cholera burden and diarrheal disease burden occurs in urban settings [20], where the increasing population density offers growing opportunities for impactful public-health interventions [21–23]. The goals of this analysis were to understand cholera dynamics in a large coastal African city and thus to help inform ministries of health and local authorities, in collaboration with international partners, when planning for the prevention of and response to cholera epidemics.

## Methods

### Population targeted by the surveillance system

A port city located on the Atlantic Ocean, Conakry is surrounded by bays and estuaries with a large part situated on a peninsula extending into the ocean. The city is divided into the municipalities of Kaloum, Dixinn, Matam, Matoto, and Ratoma, which are further divided into districts. Kaloum, Dixinn, and Matam are situated on the peninsula and Ratoma and Matoto connect the peninsula to the mainland. Conakry municipalities are not equal in terms of poverty and access to clean water: Ratoma and Kaloum are the poorest areas of Conakry, but poverty is heterogeneously distributed, sometimes occurring in pockets [24, 25]. The overall population is estimated at 1.7 million with a density of 3700 per square kilometer (website: http://www.citypopulation.de/Guinea-Cities.html, last accessed 14 Jan 2016). The rainy season extends from May to November with peaks in July and August.

### Data collection

Data for the current analysis were collected from the MoH national integrated disease surveillance and response database and the Africhol database. The MoH system collected aggregate weekly number of clinical cases nationally by district, sex, age group, and outcome. Africhol surveillance in Conakry during the study period was based on active reporting of clinical cases by every health structure in charge of treating cholera cases in the city. This included the infectious disease and pediatric wards of the Donka University Teaching Hospital during non-epidemic periods. During declared epidemics two cholera treatment centres (CTCs) were opened at the Donka hospital and the Ratoma Health Care Center. Through continuous prospective Africhol surveillance we collected individual level data on: clinical symptoms; socio-demographic profiles; place of residence; clinical outcome; and behavioural risk factors for illness (risk contacts, drinking water source, and recent travel). Overall, case counts and incidences were based on MoH data while Africhol data were used to describe clinical profiles, behavioral risk factors for illness, and microbiological confirmation during this period.

The Guinean National Institute for Public Health (INSP) performed culture confirmation of suspected cases. We aimed to collect whole stool or rectal swabs from all suspected cases, however, in practice, the proportion of cases with a collected stool was low during the large 2012 outbreaks when laboratory capacity became overwhelmed. Local staff were advised to collect the first ten cases per day only*.* Samples were transported in Cary-Blair transport medium to INSP. They were then enriched in alkaline peptone water and plated on thiosulfate-citrate-bile-salt-sucrose (TCBS) agar. Characteristic yellow colonies were sub-cultured in non-selective medium. Resulting colonies were tested for oxidase and, if positive, considered confirmed and serogrouped. External quality control was performed by the National Institute of Communicable Diseases in South Africa using PCR.

In an area where no cholera had been reported, a suspected cholera case was defined as any person aged 2 years or more who developed severe dehydration or died from acute watery diarrhoea. Once a cholera epidemic had been declared, a suspected cholera case was defined as a patient aged 2 years or more who developed acute watery diarrhoea. A confirmed case was a suspected case that tested positive for *Vibrio cholerae* by stool culture.

For the denominator, we estimated district populations during 2011–2013 by applying annual national population growth rates as provided by the MoH to the latest available census from 1996. Due to the uncertainty regarding the degree to which populations remained stable across municipalities and districts, populations and calculated attack rates should be considered approximated.

### Analysis

For the current analysis, we analysed data from August 2011 to December 2013. We defined three periods relative to the 2012 outbreak. The pre-epidemic period ran from August 1, 2011 to May 28, 2012. The epidemic period started with the official declaration of the outbreak in Conakry on May 29, 2012 (week 22) and lasted until November 4 (week 44) when Conakry reported four consecutive weeks without a clinical case. The post-epidemic period ran from November 5, 2012 to December 31, 2013.

We conducted descriptive analyses and checked if cholera prevalence was associated with various variables such as potential risk factors (primary water source, water treatment, or risky contacts) or clinical characteristics using prevalence ratios. For the epidemic period we carried out a spatial descriptive analysis and calculated weekly attack rates (AR) and AR over the whole epidemic period at district and municipal levels to describe outbreak dynamics. Significance level was 0.05. In addition, we looked for temporal-spatial clustering using Kulldorf’s SaTScan at district level to identify core clusters and calculated the standardized morbidity ratio (SMR) at district level with the ratio of observed to expected clinical cases using the entire geographic area of Conakry as the reference [26, 27]. We reclassified case residence locations to the historical district boundaries to allow comparisons with previous outbreaks. Some contiguous districts were grouped because of the lack of precision concerning the places of residence. Two districts of the Kaloum municipality, Fotoba and Kassa, are located on Loos islands, seven kilometres off the mainland shore of Conakry, and thus were not included in our analysis. These methods reduced the 97 administrative districts of Conakry to 54 districts that were used for analysis.

Statistical and spatial analyses were carried out on Stata 12.0, R 3.1.2 and SaTScan software. Maps were generated using R 3.1.2 and ArcGIS 10. The used R packages were maptools and rgdal.

## Results

**Fig. 1:**
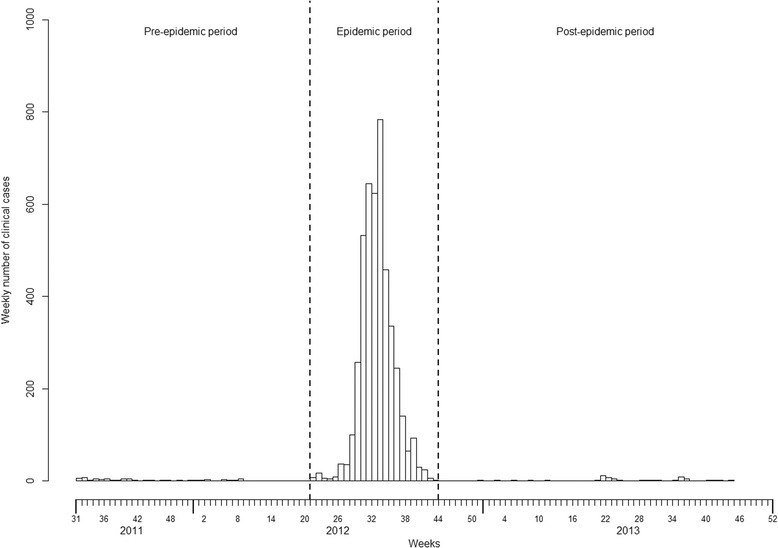
Epidemiologic Curve Describing the 2012 Cholera Epidemic in Conakry, Guinea

A total of 4559 suspected cases were reported, including 66 during the pre-epidemic, 4437 during the epidemic, and 56 during the post-epidemic periods (Fig. 1) (Table 1). All municipalities were affected, but the municipal distribution varied across periods. Two municipalities, Matoto and Ratoma, consistently reported most cases, while Kaloum reported the least. The overall AR for the epidemic period was 0.28%, with little variation across municipalities from 0.16% to 0.31%, the lowest in Kaloum and the highest in Matoto and Ratoma. During the pre-epidemic period, all 66 cases had a culture and four (6%) were positive for *V. cholerae*; during the post-epidemic period 20 cases (36%) had a culture and 14 (70%) were positive for *V. cholerae*, serotype Ogawa. Conversely, due to the overwhelmed health system, during the epidemic period 118 cases (3%) had a culture, and 71 (60%) were positive.

**Table 1:** Cholera Case Counts and Attack Rates over the Pre-Epidemic, Epidemic, and Post-Epidemic Periods by Municipality and District; Conakry, Guinea, August 2011 to December 2013^a^

District by Municipality	District population	Pre-epidemic period		Epidemic period		Post-epidemic period	
		Cases	Attack rate (%)	Cases	Attack rate (%)	Cases	Attack rate (%)
DIXINN	174 184	13	0.007	460	0.26	7	0.004
Cameroun	7803	0	0.00	10	0.13	0	0.00
Camayenne	19 927	0	0.00	22	0.11	0	0.00
Landreah	8432	0	0.00	21	0.25	0	0.00
Hafia	29 752	1	0.00	153	0.51	0	0.00
Belle Vue	26 625	0	0.00	39	0.15	0	0.00
Kenien	13 859	0	0.00	23	0.17	2	0.01
Dixinn	61 212	12	0.02	183	0.30	5	0.01
Miniere	6574	0	0.00	9	0.14	0	0.00
KALOUM	99 785	1	0.001	159	0.16	1	0.001
Tombo	10 231	0	0.00	12	0.12	0	0.00
Boulbinet	9847	0	0.00	24	0.24	1	0.01
Teminetaye	4983	0	0.00	3	0.06	0	0.00
Manquepas	12 692	0	0.00	19	0.15	0	0.00
Sans Fil	8821	0	0.00	22	0.25	0	0.00
Almamya	13 154	0	0.00	5	0.04	0	0.00
Koulewondy	6122	0	0.00	8	0.13	0	0.00
Coronthie	20 583	0	0.00	42	0.20	0	0.00
Sandervalia	13 352	0	0.18	24	0.18	0	0.00
MATAM	229 426	9	0.004	532	0.23	2	0.001
Coleah	33 645	0	0.18	59	0.18	0	0.00
Madina	33 175	0	0.24	80	0.24	0	0.00
Touguiwondy	11 698	0	0.24	28	0.24	0	0.00
Lansebounyi	16 347	0	0.07	11	0.07	0	0.00
Carriere	23 131	0	0.20	47	0.20	0	0.00
Mafanco	13 212	0	0.20	27	0.20	0	0.00
Boussoura	10 329	0	0.13	13	0.13	0	0.00
Hermakono	20 971	0	0.19	40	0.19	0	0.00
Matam	33 174	0	0.36	120	0.36	0	0.00
Bonfi	33 744	0	0.32	107	0.32	2	0.01
MATOTO	571 117	21	0.004	1720	0.30	4	0.001
Gbessia	96 835	0	0.00	329	0.34	1	0.00
Dabompa	26 568	0	0.00	105	0.40	1	0.00
Yimbaya	52 358	1	0.00	243	0.46	0	0.00
Tombolia	65 158	0	0.00	90	0.14	0	0.00
Behanzin	16 804	0	0.00	7	0.04	0	0.00
Sangoya	49 168	0	0.00	122	0.25	0	0.00
Camp Alpha YD	10 840	0	0.00	4	0.04	0	0.00
Kissosso	40 331	0	0.00	261	0.65	1	0.00
Dar Es Salam_m	17 015	0	0.00	34	0.20	0	0.00
Simbaya	47 512	0	0.00	106	0.22	0	0.00
Matoto	55 456	0	0.00	82	0.15	0	0.00
Tanene	30 866	0	0.00	101	0.33	0	0.00
Dabondy	62 206	0	0.00	236	0.38	1	0.00
RATOMA	511 084	22	0.004	1566	0.31	42	0.008
Nongo	22 971	0	0.00	166	0.72	3	0.01
Wanindara	35 486	0	0.00	69	0.19	0	0.00
Lambandji	15 034	0	0.00	233	1.55	1	0.01
Hamdallaye	74 464	0	0.00	73	0.10	0	0.00
Kobaya	5843	0	0.00	33	0.56	0	0.00
Kaporo	49 599	0	0.00	162	0.33	1	0.00
Dar Es Salam_r	37 237	0	0.00	194	0.52	0	0.00
Taouyah	14 801	0	0.00	38	0.26	0	0.00
Kipe	15 582	0	0.00	60	0.39	0	0.00
Ratoma	32 002	2	0.01	62	0.19	13	0.04
Simbaya Gare	73 014	0	0.00	94	0.13	0	0.00
Koloma	91 445	0	0.00	205	0.22	1	0.00
Sonfonia	14 438	0	0.00	118	0.82	0	0.00
Yattayah	29 168	0	0.00	59	0.20	0	0.00

^a^Sum of district case counts do not necessarily equal municipality case counts because in some instances data were not available on residence by district

No difference existed between the three periods in terms of sex ratio (female/male). Mean age differed between the epidemic period and the pre-epidemic and post-epidemic periods, 26.5 years versus 22.4 and 23.1 years (*P* = 0.014), respectively. During the pre-epidemic, epidemic, and post-epidemic periods there were five, 13, and four deaths, resulting in CFRs of 7.6%, 0.3%, and 7.1% (*P* < 0.001).

**Fig. 2:**
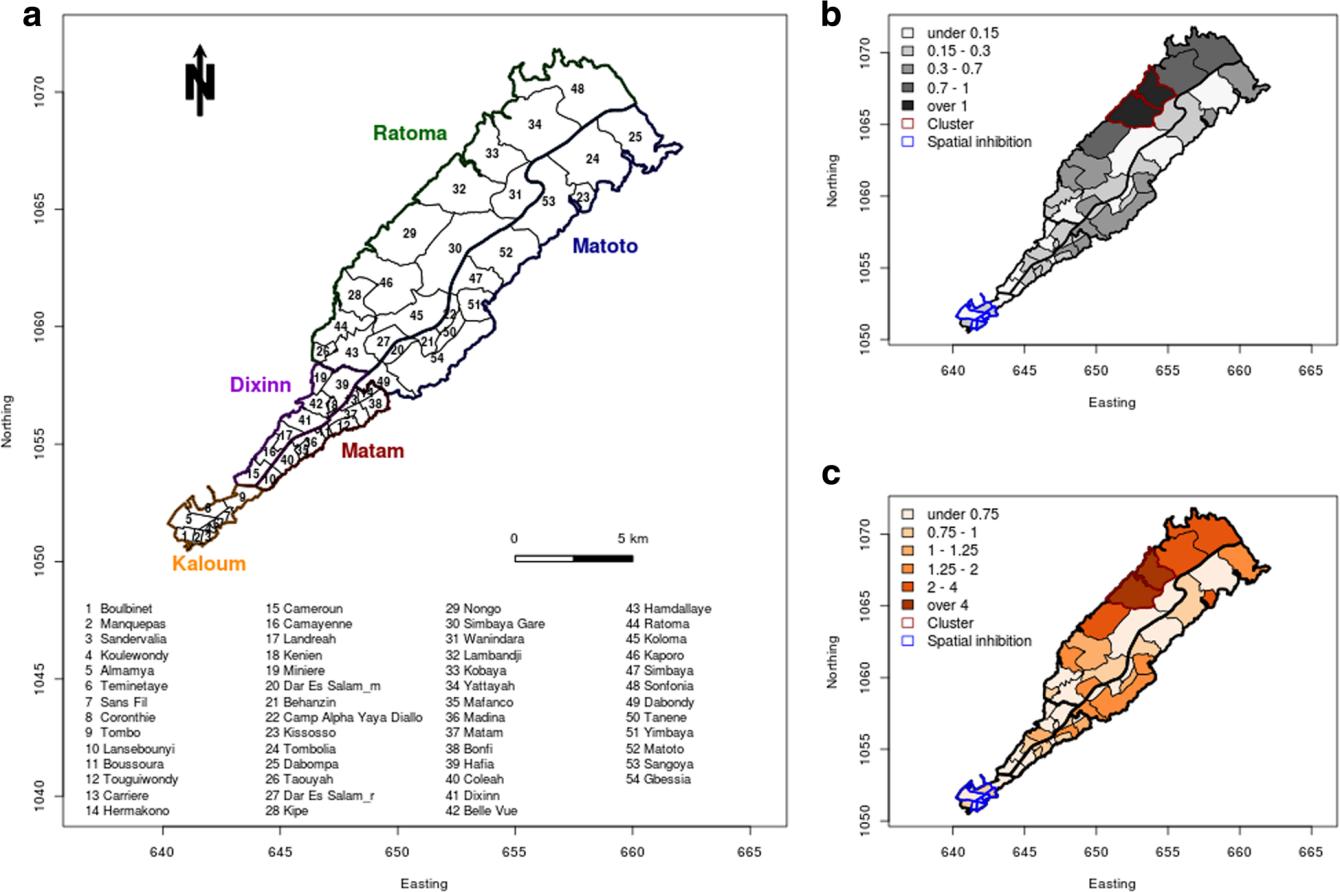
**a** List of Municipalities and Associated Districts in Conakry, Guinea, with (**b**) associated Clinical Cholera Attack Rates in Percent at District Levels during the Cholera Epidemic Period and (**c**) Cholera Standardized Mortality Ratios during the Cholera Epidemic Period, August 2011 to December 2013

At district level, ARs were heterogeneous. The highest ARs were in five contiguous districts of the northern band of Ratoma and Nongo to Sonfonia (0.72%, 1.33%, 1.21%, 0.74%, and 0.82%) as was also true for SMR (2.7, 4.9, 4.5, 2.7, and 3.0) (Fig. 2a and b) (Table 1). The SaTScan confirmed this heterogeneity and detected one cluster and one spatial inhibition zone (Fig. 2a). The cluster included two districts in the northern part of Ratoma, from week 30 to 37, and had a ratio of observed over expected number of cases of 13.1 (*P* < 0.001). This cluster was present during 8 weeks, 35% of the epidemic period, during which 87% of all cases were reported. The spatial inhibition zone included several districts at the tip of the peninsula, in Kaloum, from week 22 to 30, with a ratio of observed over expected number of cases of 0.023 (*P* < 0.001).

The symptoms of clinical cases changed over time (Table 2) with less frequent rice water stools during the pre-epidemic period and a more severe clinical profile before and after the epidemic. Some behavioural risk factors for illness also changed over time, including the primary source of drinking water and the practice of treated drinking water. A history of recent travel was also more frequent during the post-epidemic period.

**Table 2:** Clinical Profile and Risk Factors over the Pre-Epidemic, Epidemic, and Post-Epidemic Periods in Africhol Surveillance Data in Conakry, Guinea from 2011 to 2013

	Pre-epidemic period		Epidemic period	Post-epidemic period	
	Number(%)	PR^a^ (95% *CI*)	Number(%)	Number(%)	PR^a^ (95% *CI*)
CLINICAL SYMPTOMS					
Diarrhea quality					
Watery	63/65 (97%)	1.0 (1.0–1.1)	1143/1232 (93%)	34/39 (87%)	0.94 (0.83–1.1)
Rice water	9/65 (14%)	0.18 (0.10–0.33)	942/1208 (78%)	32/38 (84%)	1.1 (0.94–1.2)
Bloody	0/63 (0)	0	10/1229 (0.8%)	0/39 (0)	0
Mucous	9/64 (14%)	10 (4.7–22)	17/1226 (1.4%)	5/39 (13%)	9.3 (3.6–24)
Vomiting	60/65 (92%)	1.1 (0.97–1.1)	1062/1203 (88%)	33/38 (87%)	0.98 (0.87–1.1)
Dehydration	61/65 (94%)	3.0 (2.7–3.4)	369/1194 (31%)	33/37 (89%)	2.9 (2.5–3.3)
Dyspnea	3/62 (4.8%)	1.9 (0.60–6.1)	30/1181 (2.5%)	5/36 (14%)	5.5 (2.3–13)
Altered consciousness	9/64 (14%)	2.3 (1.2–4.4)	72/1184 (6.1%)	11/36 (31%)	5.0 (2.9–8.6)
Coma	2/64 (3.1%)	7.5 (1.5–38)	5/1195 (0.4%)	1/35 (2.9%)	6.8 (0.82–57)
PRIMARY WATER SOURCE					
Piped	13/65 (20%)	0.97 (0.59–1.6)	294/1207 (21%)	2/39 (5.1%)	0.25 (0.06–0.96)
Public tap	40/65 (62%)	0.86 (0.70–1.0)	868/1207 (72%)	15/39 (39%)	0.53 (0.36–0.80)
River/shallow well/lake	1/65 (1.5%)	0.48 (0.07–3.4)	39/1207 (3.2%)	21/39 (54%)	17 (11–25)
Other	11/65 (17%)	4.0 (2.2–7.3)	51/1207 (4.2%)	1/39 (2.6%)	0.61 (0.08–4.3)
WATER TREATMENT					
Drinking treated water	6/61 (9.8%)	0.31 (0.14–0.66)	358/1111 (32%)	3/35 (12%)	0.37 (0.13–1.1)
If treated, treated with chlorine	6/6 (100%)	1.0 (0.89–1.2)	331/342 (97%)	2/2 (100%)	1.0 (0.81–1.3)
RECENT BEHAVIORAL RISK FACTORS					
Contact with suspected cholera case	1/64 (1.6%)	0.08 (0.01–0.53)	241/1167 (21%)	4/39 (10%)	0.50 (0.19–1.3)
Funeral participation	0 (0)	0	18/1196 (1.5%)	0/39 (0)	0
Social gathering participation	3/64 (4.7%)	1.0 (0.32–3.1)	56/1195 (4.7%)	1/39 (2.6%)	0.55 (0.08–3.9)
Visited market	6/65 (9.2%)	0.37 (0.17–0.80)	294/1185 (25%)	2/39 (5.1%)	0.21 (0.05–0.80)
Travel	3/65 (4.6%)	1.8 (0.57–5.9)	30/1191 (2.5%)	5/39 (13%)	5.1 (2.1–12)

^a^Prevalence ratios (PR) and 95% confidence intervals (*CI*) were calculated for the pre-epidemic and post-epidemic periods using the epidemic period as the reference

## Discussion

The 2012 outbreak was the largest since 2007 and the current analysis has provided insights into the evolution of this event. Epidemiologically, the outbreak clustered over space and time, with attack rates of approximately 1% in the five most affected districts and most cases concentrated over a 10-week period coinciding with the peak of the rainy season. The districts of the northern part of Ratoma were among the districts with the highest AR during the 2007 outbreak, and the districts in this cluster were identified as hotspots for at least two of the three previous outbreaks [23]. The recurrent high AR and SMR likely reflect a complex situation combining environmental risks, population mobility, and behavioural characteristics [28, 29]. This northern band was identified as vulnerable due to poor water access in previous studies [24, 30]. This poor access probably results from the relatively chaotic influx and settlement of migratory rural populations, a common feature in the Ratoma area. This zone also has frequent visitation by migratory artisanal fishermen from Guinea and elsewhere who move seasonally along the coast. Within Conakry, Ratoma’s estuaries are used for agriculture, and these estuaries could be exposed to overflowing informal latrines, particularly during the rainy season [24, 31–34]. Additional issues that may contribute to cholera transmission or severity include poor food security and reduced access to health structures [24].

Our analysis also demonstrated that clinical presentation over the course of an outbreak does not remain static. In Guinea, cases during the pre-epidemic and post-epidemic periods had more severe symptoms, such as dehydration, altered consciousness, and coma. This may reflect access to healthcare as without an officially declared outbreak, care was not free, which would prompt the mainly severe cases to visit a health centre. Another feature was the less common occurrence of rice water stools during the pre-epidemic phase, likely due to a mix of etiologies, a hypothesis supported by the infrequent confirmation of *V. cholerae* during this period. Nonetheless, the mere existence of confirmed cases of cholera should raise the level of alert and preparedness among local health authorities to prevent the eruption of large outbreaks like the one we describe here.

Behavioural risk factors for cholera infection also changed over time. There was clearly a lower risk of exposure to a suspected case during the pre-epidemic phase, lower reported market attendance during the pre-epidemic and post-epidemic phases, and a large increase in use of informal rather than public water sources during the post-epidemic period. Taken together with the epidemiological and clinical results, these data suggest at least one coherent explanation for the outbreak. In brief, cholera circulation was present in Conakry well before the outbreak, at low levels and in parallel with other diarrhoea etiologies. Following this, cholera transmission via person-to-person transmission and contamination of public drinking water sources amplified the outbreak in a susceptible population cantered around Ratoma, with cholera cases overwhelming all other diarrhoea etiologies. This matches findings from nation-wide studies during the same year in Guinea [15]. Following public health interventions (such as water chlorination), acquisition of sufficient population immunity, and possibly switching away from public water sources, during the post-epidemic period, the situation gradually returned to a baseline of modest cholera circulation. Cities such as Conakry should establish routine systems for microbial risk assessment to monitor the circulation of pathogens like *V. cholerae* in the environment and guarantee the safety of drinking water sources.

Questions remain, including for example, why the 2012 outbreak did not occur earlier, since the conditions for spread did not change dramatically from 2011 to 2012. It also remains unclear why cholera has circulated for so long in Conakry, including whether an environmental reservoir exists and if so where. DNA sequencing analysis of strains isolated during the three periods is ongoing to assess their relationship with each other.

Regardless, Ratoma seems an obvious target area for efforts to reduce cholera morbidity in Conakry. Citywide, and eventually nationwide, improvements in water, sanitation, and hygiene is the optimal goal, however this is likely to take decades to achieve. A shorter-term option is the use of oral cholera vaccine. During the 2012 outbreak, Guinea implemented mass vaccination campaigns against cholera, but in the Boffa and Forecariah prefectures rather than Conakry [35], with calculated vaccine effectiveness of 87% after two doses. Vaccine was not used in Conakry due to the late phase of the outbreak. Real-time weekly analyses such as those done for the current manuscript could guide targeted, reactive vaccine interventions that might have an exaggerated impact on blunting outbreak progression. Ongoing surveillance in Conakry, and particularly in known hotspots such as Ratoma, could provide data to determine whether preventive vaccination in the region would be appropriate.

Our data, however, had several limitations. The accuracy of clinical diagnoses likely changed over time and under-reporting was more likely to have occurred at the beginning of the study period, making it difficult to have a robust comparison between the three periods we analysed. Africhol data was not comprehensive across Conakry, and thus data on behavioural risk factors and culture positivity may not be representative. Due to the nature of surveillance, no individual data was collected in the population making the comparison of cases and non-cases over time impossible, hence limiting the level of evidence we provided concerning the potential transmission routes. The population figures we used were estimates resulting from yearly growth factors applied to 1996 census data and thus attack rates should be considered rough approximations. Nevertheless, the most recent 2012 population estimates demonstrated roughly similar values for municipalities (2012 data were not available at district level, and district boundaries had also changed since the previous population estimates). No precise data was available on water and sanitation interventions carried out during the 2012 outbreak. Such interventions were performed by MoH partners, such as Action contre la Faim (ACF) or Médecins Sans Frontières (MSF), and could have had an impact on the pattern we observed. Moreover, data on consumption of food at risk was not collected either preventing us from considering the contribution of other potential transmission routes. The SaTScan is sensitive to the proximity of districts bordering areas outside the study zone (with no data available) and the shape of the study region; to account for this, we conducted a sensitivity analysis to identify the location and size of the core of cluster and spatial inhibition zones, which should minimize the impact of this limitation.

## Conclusions

Given Conakry’s poor water and sanitation systems [31] and ongoing cholera circulation either from an environmental reservoir or continuous reintroduction, the introduction of susceptible populations and the waning immunity of resident populations will most likely result in recurrent outbreaks. Yet, through the present analysis we can show how the public health challenges that seem initially overwhelming and ubiquitous are largely clustered around one district of the city. With that perspective, the problem becomes much more manageable. As a hub city, outbreaks in Conakry have the potential to spread nationally and regionally. In addition, the situation in Conakry reflects that of other large coastal West African cities - unplanned and rapid urbanization, concentrations of recent immigrants into densely populated areas, and a lack of clean water and basic sanitation [24, 29]. The current collaboration between the Guinean MoH, non-governmental organizations, and the Africhol Network should help provide the data necessary to develop targeted, efficient interventions in the short term and promote better public health infrastructure. As governments in cholera-prone countries have to establish sustainable systems for assuring safe water and sanitation for their population, prioritizing high-incidence areas like Ratoma should rapidly bring efficient results.

## Additional file

Additional file 1: Multiligual abstracts in the five official working languages of the United Nations. (PDF 501 kb)

## Abbreviations

Africhol: African Cholera Surveillance Network
AR: Attack rate
CFR: Case fatality ratio
CTC: Cholera treatment center
INSP: Guinean National Institute for Public Health
MoH: Ministry of health
SMR: Standardized morbidity ratio
