# Supplementary material for: Temporo-spatial dynamics and behavioural patterns of 2012 cholera epidemic in the African mega-city of Conakry, Guinea
Source: Infect Dis Poverty. 2018 Feb 15;7:13. doi: 10.1186/s40249-018-0393-8 (PMC5815196; doi:10.1186/s40249-018-0393-8)
Supplement: Supplementary file 1 — Multiligual abstracts in the five official working languages of the United Nations. (PDF 501 kb) [file 40249_2018_393_MOESM1_ESM.pdf]

Translation of the abstract into the five official working languages of the United Nations

الديناميات الزمنية المكانية والأنماط السلوكية خلال وباء الكوليرا عام 2012 في مدينة كوناكري الأفريقية الضخمة، بغينيا

ألكسندر بليك، وفيرونيك سار كيتا، ودلفين سوفيجوت، ومامادو ساليو، وبيرتي ماري نجانبوب، وفود سوري، وبرتراند سودري، وكويفوغوي لامين، ومارتن منجيل، وبرادفورد د. جيسنر، وكيتا ساكوبا

نبذة مختصرة:

خلفية: تتوطن الكوليرا في غينيا، حيث عانت من تفشيها المتواصل من عام 2004 إلى عام 2008، ثم هدأت إلى أن تفشى وباء عام 2012. نحن نصف هنا الخصائص الزمنية المكانية والسلوكية لحالات الكوليرا في كوناكري خلال فترة ثلاث سنوات، بما في ذلك وباء عام 2012 الكبير.

الأساليب: لقد استخدمنا بيانات المراقبة لشبكة مراقبة الكوليرا الأفريقية والوطنية (أفريتشول) التي جُمعت من جميع مراكز علاج الكوليرا في مدينة كوناكري من أغسطس 2011 إلى ديسمبر 2013. وقد وُصف انتشار حالات الكوليرا المشتبه بها والثابتة، ونسبة الوفيات الناجمة عن الإصابة، والعوامل المرتبطة بالاشتباه في الكوليرا وفقًا لثلاث فترات: فترة ما قبل الوباء (ألف)، وفترة وباء 2012 (باء)، وفترة ما بعد الوباء (جيم). ولقد حُسبت معدلات الإصابة الأسبوعية والتجمعات الزمنية المكانية على مستوى البلديات للفترة باء. وثبتت حالات الكوليرا بإجراء المزرعة في المختبر المرجعي الوطني للكوليرا.

النتائج: لقد أُبلغ عن مجموع 559 4 حالة مشتبه بها: وهي 66 و4 437 و66 حالة مشتبه بها في الفترات ألف وباء وجيم على التوالي. وثبت من بين 204 حالة مشتبه بها من نتائج المزرعة المتوفرة، إصابة 6٪، و60٪، و70٪ في الفترات ألف وباء وجيم، على التوالي. وبظهور نتيجة 0.3٪، كان معدل الوفيات بسبب المرض أقل بكثير في الفترة باء من الفترات ألف (7.6٪) وجيم (7.1٪). وكان معدل الإصابة الإجمالي 0.28٪ في الفترة باء، وتتراوح بين 0.17٪ إلى 0.31٪ بين البلديات. وفي الوقت نفسه، تحددت مجموعة من الحالات في مقاطعتين في الجزء الشمالي من كوناكري. وبالنتيجة 14٪، كان البراز الشبيه بماء الأرز أقل تواترا في الفترة ألف من الفترة باء وجيم (78٪ و84٪).

وكان الجفاف (31٪ مقابل 94٪ و89٪) والغيبوبة (0.4٪ مقابل 3.1٪ و2.9٪) أقل خلال الفترة باء من الفترتين ألف وجيم. وكانت معالجة مياه الشرب أقل تواترًا في الفترة ألف، كما وجدت المزيد من التقارير عن السفر مؤخرًا في الفترة جيم.

الاستنتاجات: لقد كشفت دينامية الوباء والوصف الاجتماعي للحالات المشتبه فيها قبل الوباء الضخم وخلاله وبعده أن الكوليرا الضمة موجودة بالفعل قبل الوباء. ومع ذلك، يبدو أن الأفراد المصابين يتفاعلون بشكل مختلف من حيث شدة المرض، ووصولهم إلى المياه المعالجة، وعادات السفر. وينبغي إجراء مثل هذا الوصف المتعمق لأوبئة الكوليرا بصورة منهجية في البيئات المتوطنة بالكوليرا من أجل إعطاء الأولوية للمناطق ذات المخاطر العالية، وتحديد عوامل الانتقال، وتحسين التدخلات الوقائية.

Translated from English version into Arabic by Zabraa_Hesham, through

TRANSLATORS
WITHOUT BORDERS

Translation of the abstract into the five official working languages of the United Nations

几内亚科纳克里 2012 年霍乱流行期间霍乱病例的时空动力学和行为模式

Alexandre Blake, Veronique Sarr Keita, Delphine Sauvageot, Mamadou Saliou, Berthe Marie Njanpop, Fode Sory, Bertrand Sudre, Koivogui Lamine, Martin Mengel, Bradford D. Gessner and Keita Sakoba[2]

摘要：
引言：霍乱是几内亚流行，2004-2008 年连续暴发，直到 2012 年又重新流行。本文描述了包括 2012 年大规模流行在内的三年期间科纳克里霍乱病例的时空和行为特征。
方法：使用 2011 年 8 月至 2013 年 12 月期间采自科纳克里各霍乱治疗中心的国家和非洲霍乱监测网络(Africhol)的监测数据，描述三个时期：流行前（A）、2012 年流行期间（B）和流行后（C）的疑似和确诊霍乱病例的患病率、病死率和疑似霍乱有关的因素。在市级水平上计算 B 期的每周发病率和时间-空间的聚集性。通过在国家霍乱参考实验室进行增菌分离培养以确定霍乱确诊病例。
结果：共报告 4559 例疑似病例，其中，A、B、C 期分别有 66 例、4437 例和 66 例。在有培养结果的 204 例疑似病例中，在 A、B、C 期分别有 6%、60%和 70%被确诊。B 期病死率为 0.3%，显著低于 A 期(7.6%)和 C 期(7.1%)。B 期的总发病率为 0.28%，而各市的发病率范围为 0.17-0.31%。同时，在科纳克里北部的两个地区发现了一组病例。A 期的米泔水样便比例为 14%，低于 B 期的 78%和 C 期的 84%。B 期脱水率（31%）和昏迷率(0.4%)均低于 A 期和 C 期的脱水率（94%，89%)和昏迷率（3.1%，2.9%）。A 期的饮用水处理频率较低，而 C 期则有较多近期旅行的报告。
结论：在大规模疫情发生前、期间和发生后，对疑似病例的流行动态和社会学描述揭示了霍乱弧菌在流行之前就已存在。然而，似乎感染者对疾病的严重程度以及接受处理过的水和旅行习惯有不同的反应。应该在霍乱流行地区系统地进行霍乱疫情的深入分析，以确定高风险地区的优先次序、传播因素和优化预防措施。

Translated from English version into Chinese by Translated by Xue-Jiao Teng, edited by Pin Yang

CHINESE CENTER FOR DISEASE CONTROL AND PREVENTION
NATIONAL INSTITUTE OF PARASITIC DISEASES

Dynamique spatiotemporelle et schémas comportementaux au cours de l'épidémie de choléra de 2012 dans la mégalopole africaine de Conakry, en Guinée

Alexandre Blake, Véronique Sarr Keita, Delphine Sauvageot, Mamadou Saliou, Berthe Marie Njanpop, Fode Sory, Bertrand Sudre, Koivogui Lamine, Martin Mengel, Bradford D. Gessner and Keita Sakoba[2]

Résumé
Contexte : Le choléra est endémique en Guinée, pays qui a connu des épidémies successives de 2004 à 2008, puis un répit avant celle de 2012. Nous décrivons ici les caractéristiques spatiotemporelles et comportementales des cas de choléra à Conakry sur une période de trois ans qui inclut la vaste épidémie de 2012.
Méthodes : Nous avons utilisé les données de surveillance du réseau national de surveillance du choléra et du réseau africain Africhol, recueillies dans tous les centres de traitement du choléra de Conakry entre août 2011 et décembre 2013. La prévalence des cas suspects et confirmés de choléra, le taux de mortalité des cas (CFR) et les facteurs associés aux cas suspectés ont été décrits sur trois périodes : avant (A), pendant (B) et après (C) l'épidémie de 2012. Les taux de déclaration hebdomadaires et l'agrégation spatiotemporelle ont été calculés au niveau des municipalités pour la période B. Le choléra était confirmé par culture au laboratoire de référence du choléra.
Résultats : Au total, 4559 cas suspects ont été rapportés : 66, 4437 et 66 pendant les périodes A, B et C, respectivement. Sur les 204 cas suspects pour lesquels des résultats de culture sont disponibles,

6 %, 60 % et 70 % ont été confirmés, respectivement, pendant les périodes A, B et C. À 0,3 %, le CFR était significativement plus bas pendant la période B que pendant les périodes A (7,6 %) et C (7,1 %). Le taux de déclaration global était de 0,28 % pendant la période B, avec des extrêmes de 0,17 % à 0,31 % selon les municipalités. Dans le même temps, un cluster de cas a été identifié dans deux districts des quartiers nord de Conakry. Les selles en eau de riz étaient moins fréquentes pendant la période A (14 %) que pendant les périodes B (78 %) et C (84 %). La déshydratation (31 % contre 94 % et 89 %) et le coma (0,4 % contre 3,1 % et 2,9 %) étaient moins fréquents pendant la période B que pendant les périodes A et C. Le traitement de l'eau potable était moins fréquent pendant la période A et il y a eu moins de voyages récents rapportés pendant la période C.
Conclusions : La dynamique de l'épidémie et la description sociologique des cas suspects avant, pendant et après la grande épidémie ont révélé que *Vibrio cholerae* était déjà présent avant celle-ci. Il s'est cependant avéré que les individus réagissaient différemment, aussi bien en termes de gravité de la maladie que d'accès à de l'eau traitée et d'habitudes de voyage. Cette description approfondie d'une épidémie de choléra serait systématiquement nécessaire dans les régions de choléra endémique, afin d'identifier les zones à haut risque prioritaires, d'identifier les facteurs de transmission et d'optimiser les interventions préventives.

Translated from English version into French by Suzanne Assenat, through

TRANSLATORS
WITHOUT BORDERS

Пространственно-временная динамика и модели поведения во время эпидемии холеры 2012 года в африканском городе-гиганте Конакри, Гвинея

Александр Блейк, Вероника Сарр Кеита, Дельфина Соважо, Мамаду Салиу, Берта Мари Ньяпо, Фод Сори, Бертранд Судр, Куавогуи Ламин, Мартэн Менгель, Бредфорд Д. Гесснер и Кеита Сакоба[2]

Аннотация:
История вопроса: Холера является эндемическим заболеванием в Гвинее, последовательные вспышки которого наблюдались с 2004 по 2008 год, сопровожденные затишьем до наступления эпидемии в 2012 году. В статье описываются пространственно-временные и поведенческие характеристики случаев заболеваний холерой в Конакри во время трехлетнего периода, включая крупномасштабную эпидемию 2012 года.
Методы: Мы использовали данные наблюдений национальной и Африканской сети наблюдения за холерой (Africhol), полученные от каждого медицинского центра заболеваний холеры в городе Конакри с августа 2011 по декабрь 2013 года. Распространение подозрительных и подтвержденных случаев холеры были описаны согласно трем периодам: предэпидемическому (А), эпидемическому (Б) и постэпидемическому (В). Еженедельные показатели пораженности и пространственно-временное скапливание случаев холеры были рассчитаны на муниципальном уровне за период Б. Холера была подтверждена культурой в национальной справочной лабораторией.
Результаты: Всего поступило 4559 сообщений о подозрительных случаях: 66, 4437 и 66 в период А, Б и В соответственно. Среди 204 подозрительных случаев с доступными результатами культуры 6%, 60% и 70% были подтверждены в периоды А, Б и В соответственно. Летальность с показателем 0,3% была значительно ниже в период Б, чем в период А (7,6%) и В (7,1%). Общий показатель пораженности был 0,28% в период В и колебался в пределах от 0,17% до 0,31% на территории муниципалитетов. Одновременно скопление случаев было выявлено в двух округах в северной части Конакри. На уровне 14%, стул в виде рисового отвара был менее частым в период А, чем в периоды Б и В (78% и 84%). Обезвоживание (31% в сравнении с 94% и 89%) и кома (0.4% в сравнении с 3.1% и 2.9%) были ниже в течение периода Б, чем в периоды А и В. Лечение питьевой водой было менее частым в период А, в то время как в период В поступало больше сообщений о недавних

путешествиях.
Выводы: Динамика эпидемии и социологическое описание подозрительных случаев до, во время и после крупномасштабной эпидемии выявили, что холерный вибрион (лат. *vibrio cholerae*) уже присутствовал до эпидемии. Однако, следует отметить, что инфицированные реагировали по-разному в зависимости от как тяжести заболевания, так и доступа к очищенной воде и привычек относительно путешествий. Такого рода подробное описание эпидемии должно быть выполнено в эндемических условиях холеры для того, чтобы отдавать приоритет районам повышенного риска, устанавливать факторы передачи заболевания и оптимизировать профилактические меры.

Translated from English version into Russian by IraKulinevych, through

TRANSLATORS WITHOUT BORDERS

Dinámicas temporales y espaciales, y patrones comportamentales durante la epidemia de cólera de 2012 en la megaciudad de Conakri, Guinea

Alexandre Blake, Veronique Sarr Keita, Delphine Sauvageot, Mamadou Saliou, Berthe Marie Njanpop, Fode Sory, Bertrand Sudre, Koivogui Lamine, Martin Mengel, Bradford D. Gessner y Keita Sakoba2

Resumen:
Contexto: Tras brotes consecutivos entre 2004 y 2008, seguidos de un periodo de calma hasta la epidemia de 2012, se puede decir que el cólera es endémico en Guinea. Aquí describiremos las características temporales, espaciales y comportamentales de los casos de cólera en Conakri durante un periodo de tres años que incluye la epidemia a larga escala de 2012.
Métodos: Hemos usado las estadísticas que se recogieron de cada tratamiento de cólera en el centro de Conakri en la Red africana para la vigilancia del cólera (Africhol) y la red nacional desde agosto de 2011 hasta diciembre de 2013. La prevalencia de casos sospechados y confirmados de cólera, la tasa de letalidad, y los factores asociados a casos sospechosos de cólera se describieron en tres periodos: pre-epidemia (A), epidemia de 2012 (B) y post epidemia (C). Las tasas de ataques semanales y los agrupamientos temporales y espaciales se calcularon a nivel local para el periodo B. El cólera se confirmó a través de cultivos en el laboratorio de referencia nacional del cólera.
Resultados: Se declaró un total de 4.559 casos sospechosos: 66, 4.437 y 66 casos sospechosos en los periodos A, B y C respectivamente. Entre los 204 casos sospechosos con resultados disponibles de cultivos, se confirmaron el 6 %, el 60 % y el 70 % respectivamente entre los periodos A, B y C. Con un 0,3 %, la tasa de letalidad durante el periodo B fue considerablemente más baja que en los periodos A (7,6 %) y C (7,1 %). La tasa total de ataques fue de 0,28 % en el periodo B, a través de los distintos distritos osciló entre 0,17% y 0,31 %. Al mismo tiempo, se identificó un grupo de casos en dos distritos en la zona norte de Conakri. Con un 14 %, las diarreas acuosas fueron menos frecuentes en el periodo A que en los B (78 %) y C (84 %). La desidratación (31 % vs. 94 % y 89%) y el coma (0,4 % vs. 3,1 % y 2,9 %) fueron más bajos en el periodo B. Beber agua como tratamiento fue menos frecuente en el periodo A, mientras que en el periodo C se declararon más viajes recientes.
Conclusiones: La dinámica de la epidemia y la descripción sociológica de los casos sospechosos antes, durante y después de la gran epidemia revelaron que el *Vibrio cholerae* ya estaba presente antes de la epidemia. Sin embargo, parece que los individuos infectados reaccionaron de maneras diferentes en lo que se refiere a la gravedad de la enfermedad, así como su acceso a agua tratada y sus hábitos de viaje. Se deberían llevar a cabo sistemáticamente descripciones así de detalladas de las epidemias de cólera para dar prioridad las áreas de riesgo más alto, identificar factores de transmisión y optimizar las intervenciones preventivas.

Translated from English version into Spanish by Berpergar, through

TRANSLATORS
WITHOUT BORDERS
